# Long-Range Proton Transfer in 7-Hydroxy-Quinoline-Based Azomethine Dyes: A Hidden Reason for the Low Efficiency

**DOI:** 10.3390/molecules27238225

**Published:** 2022-11-25

**Authors:** Michał F. Rode, Daniela Nedeltcheva-Antonova, Liudmil Antonov

**Affiliations:** 1 Institute of Physics, Polish Academy of Sciences, Aleja Lotników 32/46, 02-668 Warsaw, Poland; 2 Institute of Electronics, Bulgarian Academy of Sciences, 72 Tzarigradsko Chaussee blvd., 1784 Sofia, Bulgaria; 3 Institute of Organic Chemistry with Centre of Phytochemistry, Acad. G.Bonchev Str., bl. 9, 1113 Sofia, Bulgaria

**Keywords:** 7-hydroxyquinoline, proton crane, ESIPT, tautomerism, *ab initio* calculations

## Abstract

In the tautomeric Schiff bases, derived from 7-hydroxyquinoline, two competitive channels are possible upon excitation of the enol tautomer, namely proton transfer (PT) through intramolecular hydrogen bonding to the corresponding keto form and *trans*–*cis* isomerization around the azomethine double bond. The former leads to switching, based on twist-assisted excited state intramolecular PT, where the long-range proton transfer can occur as a targeted process. The latter, determined by the flexibility of the crane part, reduces the efficiency of the main targeted process. In previously studied molecular switches based on the 7-hydroxyquinoline skeleton, only the intramolecular PT photo-process undergoing from the excited enol form towards the keto tautomer, which is in most cases barrierless, has been discussed. Therefore, in the current study, the ground state PT properties and isomerization of (E)-8-((phenylimino)methyl)quinolin-7-ol and (E)-8-(((pentafluorophenyl)imino)methyl)quinolin-7-ol are investigated in depth using the MP2 methodology, while the excited state energy profiles are calculated with the ADC(2) method. The obtained results are discussed in light of the existing experimental data.

## 1. Introduction

Photo switching systems, based on the proton craning mechanism, are the molecules where a proton is transferred intramolecularly over a long distance within the same molecule. Called proton cranes, they contain in general a tautomeric unit (frame) with clearly defined, spatially separated, proton donor and proton acceptor sites, which exchange, under external stimuli, the tautomeric (or pseudo tautomeric) proton, using a crane unit as a proton delivery system [1,2,3,4,5,6].

7-hydroxyquinoline (**7HQ**) is one of the most frequently studied examples of long-range proton transfer (PT) [7,8,9,10,11,12,13,14,15,16,17], where the proton donating OH group and the proton accepting N atom are far apart to provide conditions for truly intramolecular proton exchange. The enol tautomer is substantially more stable, while the NH tautomer has been experimentally observed only in protic organic solvents or in the presence of water and results from the intermolecular, solvent-assisted PT mechanism [17,18]. Previous computational studies of the potential energy profiles along the relevant internal coordinates, such as the O–H and N–H bond stretching distances, suggested that forward and backward PT process could be observed for a series of molecules containing **7HQ**, as a general tautomeric frame for the location of the O and N atoms in the space, and oxazine [5,19], other six-membered ring heterocycles [19], pyridines [19], carbaldehyde groups [18], and CO-BF_2_ [20,21] as proton cranes. Among them, the smallest in size, the carbaldehyde moiety, seemed to be a reasonably good choice allowing for fast rotation even when protonated [18,22].

**Scheme 1:**
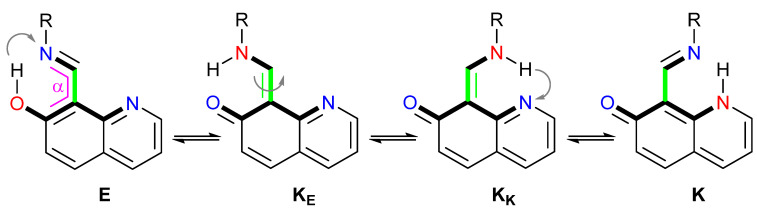
Long-range PT reaction of the proton cranes based on **7HQ** as a tautomeric sub-unit and C=N group as a crane sub-unit. The proton donor and proton acceptor sites are given in red and blue, respectively. The axle of intramolecular rotation is given in green. The twisting angle α is given in magenta.

**Scheme 2:**
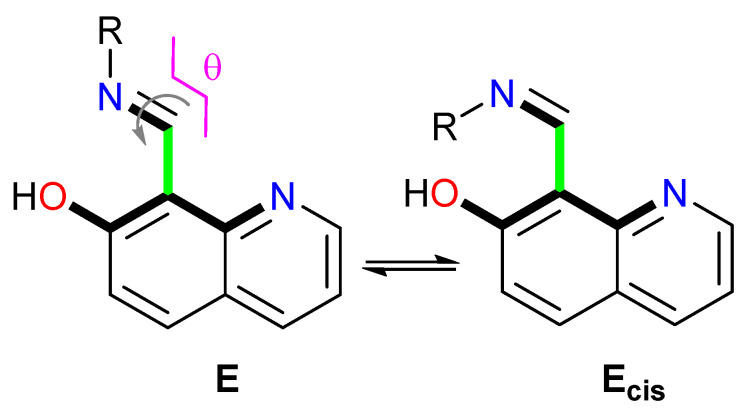
*Trans*–*cis* isomerization around the C=N bond. The twisting angle θ is given in magenta.

**Scheme 3:**
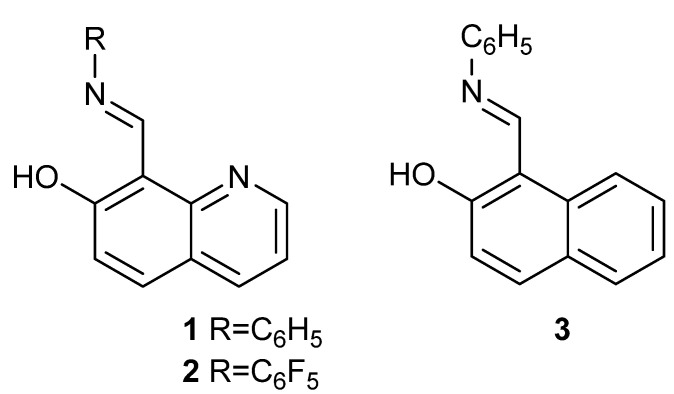
Compounds, investigated in the current study, presented in their enol tautomeric forms.

Recently, we have reported several classes of proton craning switches, based on **7HQ**, where the azomethine group plays a role of a crane unit, providing, in some cases, truly intramolecular long-range PT upon irradiation [23,24,25]. It was shown (Scheme 1) that the proton is transferred from the **7HQ** oxygen atom to the basic nitrogen through series of short-range PT steps, accompanied by intramolecular rotation around the axle binding the tautomeric and crane units. The process is initiated by photoexcitation of the **E**-tautomer, followed by excited-state intramolecular PT (ESIPT) to K_E_*, consecutive intramolecular rotation and relaxation to the ground state intermediate tautomers (**K_E_** and **K_K_**) through a conical intersection (CI) [5], leading a priori to relatively low yield of formation of the terminal form **K**. The efficiency could be additionally worsened by the potential possibility of a competitive process originating from the flexibility of the crane unit, namely *trans*–*cis* isomerization around the azomethine bond [26,27], as shown in Scheme 2. Therefore, in the current study, we investigate in depth, by using advanced theoretical calculations, the ground and excited state PT properties and isomerization of compounds **1** and **2** (Scheme 3), which are based on **7HQ**. Compound **3**, where long-range PT to **K** is impossible, is taken as a reference compound of **1**. The theoretical results will be compared with the existing experimental data in order to define simple tips allowing to distinguish between the long-range PT and the C=N *trans*–*cis* isomerization in proton cranes with a flexible crane part.

## 2. Results and Discussion

The studied **1** and **2**, belong to the class of photo switchable molecules, which photo switching mechanism is based on the twist-assisted ESIPT process [5,19]. Each of them consists of the two units in conjugation: the proton donating **7HQ** frame and the proton accepting crane (C=N) being attached to position eight of the **7HQ** tautomeric frame. As a result of photoexcitation of the enol form, (**E**), **7HQ** is able to donate the proton to the crane, while its own proton acceptor site (quinoline N atom) is far away. The proton crane is able to accept the proton in the excited state (S_1_), rotate, and dissipate photoexcitation energy to the ground state due to the internal conversion process undergoing in the vicinity of the CI(S_1_/S_0_) geometry. The CI geometry corresponds to a structure with both the crane and tautomeric frame units being perpendicular to each other, and thus both states (S_0_ and S_1_) are prone to meet leading to nonadiabatic transition S_1_→S_0_. This may result in the population of either the initial enol form, (**E**), or, the generation of a new keto form, (**K**). An experiment conveyed in a low-temperature Ar-matrix isolation proved that such a photo switching mechanism, based on single twist-assisted ESIPT, can be a reversible process gaining from enol to keto, **E**→**K**, and back, from keto to enol, **K**→ **E** with UV light excitation at a proper wavelength or thermally [22].

The reversibility of the switching process is based on the different stability of the two terminal forms, **E** and **K**. Of course, the real picture also depends on the relative stability of the intermediate tautomers **K_E_** and **K_K_** (Scheme 1). Although the aim is to have clean switching from **E** to **K**, depending on the particular molecular structure and relative stability of all four possible tautomers, the switching process can start from a mixture of tautomers and end in another tautomeric equilibrium, as it happens in **1** [24].

**Figure 1:**
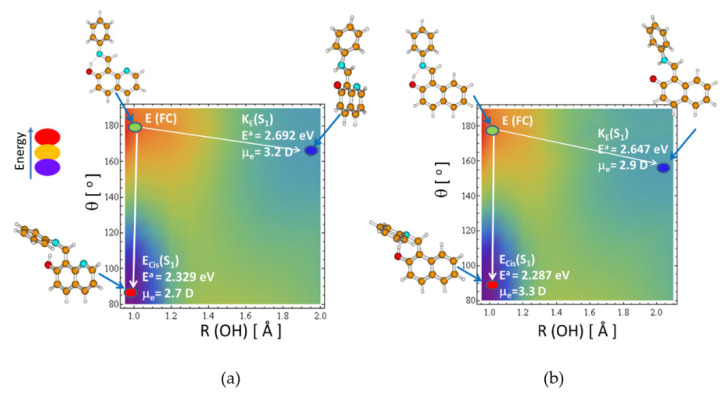
Relaxed potential energy surface of the lowest excited electronic state, S_1_, of **1** (**a**) and **3** (**b**), as a function of R(OH) stretching and θ (C = N) torsion angle coordinates, showing competition between the two processes: **E**→ **K_E_** ESIPT and **E→E_cis_** photoisomerization after photoexcitation of the S_0_-state **E** global minimum in the Franck-Condon region. The green circle represents the Franck–Condon region of the ground-state **E** global minimum initially populated after photoexcitation (**E**(FC)), the blue circle represents the excited-state **K_E_**(S_1_) proton-transferred minimum, and the red circle represents the **E_cis_**(S_1_) excited-state minimum. The results were obtained with the aid of the ADC(2)/cc-pVDZ method. S_1_-state dipole moment, *μ_e_*, in Debye. Adiabatic energy, *E^a^*, in eV.

**Figure 2:**
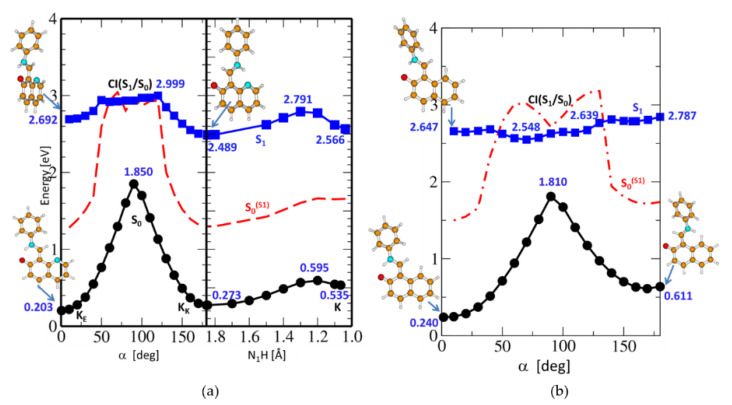
Relaxed potential energy scans of **1** (**a**) and **3** (**b**) in the S_0_ state (circles), determined at the MP2/cc-pVDZ theory level in the S_1_ state (blue squares), determined at the ADC(2)/cc-pVDZ theory level along the minimum potential energy path for N-phenyl crane torsion (**K_E_** to **K_K_**, α coordinate), and for hydrogen transfer from the intermediate form **K_K_**, toward terminal form **K** (N_1_H coordinate). S_0_^(S1)^ denotes the energy of the S_0_ state, calculated at the geometry of the S_1_ excited state (red dashed curves). Adiabatic energies (in blue), *E^a^*, in eV.

In most of the photo switchable molecules based on **7HQ,** studied so far [5,19,20,21], the ESIPT process from the initially photoexcited enol form towards the nitrogen atom in the proton crane to form proton-transferred K_E_(S_1_) tautomer was considered as a dominating channel. However, the presence of the double C=N bond in the azomethine craning group gives a possibility of an additional geometrical transformation in the molecule, namely *trans*–*cis* photoisomerization. Both are activated in the excited state just after photoexcitation of the **E**(S_0_) form (to be punctual, **E** is actually **E_trans_**, but for simplicity, only **E** will be used in the discussion below). Thus, these two excited-state processes seem to be coupled and their competition is visualized in Figure 1, where two-dimensional potential energy surfaces (PES) of the first (S_1_) exited states of **1** and **3** are shown. Each point at the PES is obtained by optimization of the geometry of the corresponding molecule imposing two constraints for driving coordinates: R(OH) stretching and θ dihedral angle (as defined in Scheme 2), separately in a given electronic state. The ESIPT process in such constructed S_1_-PES can be visualized along the direction parallel to the R(OH)-axis. The isomerization process, however, can be observed along the direction parallel to the θ-axis. The excited state potential energy surface of the next steps of the twist-assisted ESIPT process (according to Scheme 1) is shown in Figure 2.

### 2.1. Ground State Tautomerization and Isomerization

The relative stability of the ground state tautomers of **1** and **3** are summarized in Table 1 and Table 2, respectively. The same information for **2** is given in the Supplementary Materials part Table S1. 

As seen in Table 1, the enol form, **1E**, constitutes the ground state global minimum (*μ*_g_ = 3.2 D) of the compound in the gas phase. The intramolecular hydrogen bond OH…N ensures planarity of the **7HQ** ring along the C_7HQ_-C=N-C_Ph_ backbone, while the phenyl ring attached to the azomethine N atom is tilted out of the plane of the **7HQ** skeleton. The second-in-energy, **1K_E_** tautomer lying 0.203 eV (Table 1) above the **1E** global minimum is separated from it by a very low energy barrier of 0.048 eV (details are given in the Supplementary Materials part Table S2). Both isomers seem to be present in thermal equilibrium. The third-in-energy keto tautomer **1K_K_** (*E^a^*=0.274 eV) can be obtained by rotating the proton transferred **1K_E_** minimum by ~ 180^o^, about the α dihedral angle (as defined in Scheme 1). The barrier separating those two intermediate keto tautomers in the S_0_ state is huge, at ~1.6 eV, which precludes their direct S_0_-state isomerization. Putative **1K** form related to its **1K_K_** tautomer by proton transfer between the azomethine N and quinoline nitrogen atom is lying much higher in energy, by 0.535 eV, and is separated from the **K_K_**(S_0_) tautomer with an energy barrier of 0.060 eV. This is why the **K**(S_0_) tautomer is rather not likely to be populated both thermally due to the photo process. Contrary to **1K_E_**, the relatively high-in-energy **1E_cis_**(S_0_) isomer (see Table 1) lying 0.531 eV above the global minimum cannot be populated thermally due to the large S_0_-state energy barrier of 1.35 eV separating it from the **1E**(S_0_) isomer, but it can be photo populated in the reversible photoisomerization process. The S_0_-state energy barrier for the back **E_cis_**(S_0_) → **E**(S_0_) isomerization is also large, at +0.8 eV, assuming a slow thermal depopulation of **E_cis_**(S_0_). As seen in Table S1 (see see Supplementary Materials part), the fluorine substitution in **2**, leads to reduced basicity of the azomethine nitrogen atom and, hence, to a lower stability of the intermediate tautomers. The terminal tautomer **2K** is slightly stabilized, but remains higher in energy compared to **2K_K_**, which makes its population improbable. 

The theoretical data for model compound **3** (Table 2), and the existing experimental data for the ground state tautomerism of **1** [24] and **3** [28], allow us to validate the current theoretical findings. Comparing the data from Table 1 and Table 2, and bearing in mind that results for the gas phase are shown, it seems that in both compounds the stability of the **E** and **K_E_** tautomers should be similar in solution. From one side, the **3K_E_** tautomer is slightly less stable compared to **1K_E_**, but at the same time, the former is more polar compared to the corresponding enol, so the increased polarity of the solvent should compensate for this difference. The experimental results show that the ΔG value at room temperature in cyclohexane for **3** is around 1.4 kcal/mol [28], indicating a more stable **E** tautomer, as suggested by the theoretical calculations. In the case of **1** in toluene, all three (**E**, **K_E_,** and **K_K_**) tautomers are presented, again with the enol form being dominant [24]. The increased solvent polarity by using acetonitrile as a solvent shift the equilibrium towards the more polar **K_E_** in **3** and the more polar **K_K_** in **1** [24]. In **2**, the **E** form predominance and the existence of traces of **K_E_** only, are evident from the absorption and NMR spectra in agreement with the data collected in Table S1 (see Supplementary Materials part). Therefore, we can assume that the used level of theory correctly predicts the tendencies in the ground state energy landscape, bearing in mind that the MP2 theory overestimates the stability of the enol tautomer, as previously observed [28]. 

### 2.2. ESIPT vs. Trans–cis Isomerization in *S_1_*

**Figure 3:**
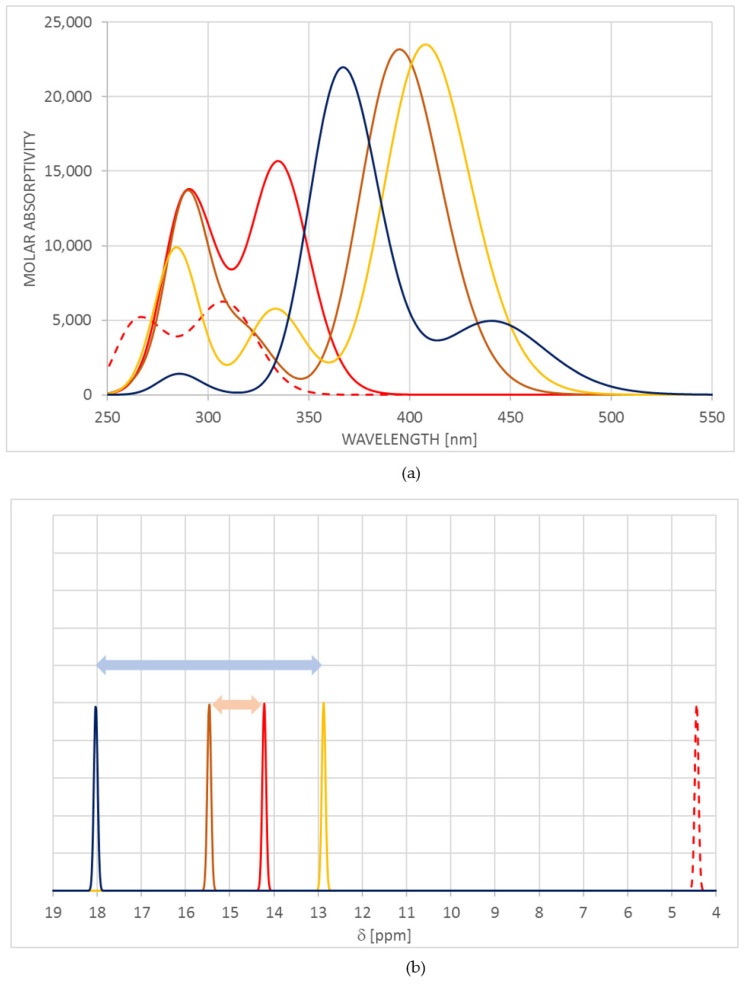
(**a**, top) Simulated absorption spectra of **1**, using the data from Table 1; (**b**, down). Simulated ^1^H NMR chemical shifts of the tautomeric proton. The tautomeric form is given as follows: **E**—red solid line, **K_E_**—brown solid line, **K_K_**—yellow solid line, **K**—blue solid line, and **E_cis_**—red dashes.

In the calculated absorption spectrum of the global minimum, **E**(S_0_), there are three lowest strongly absorbing states: S_0_→S_1,2,3_ of meaningful oscillator strength (see Table 1 and Table 2, and Figure 3). S_0_→S_1_ is the most intense transition for all three compounds with a maximum absorption slightly blue-shifted for **3** vs. **1** and **2**. Since the photo physics of **1** and **2** seems to be similar (see Figure 2a and Figure S1 (see Supplementary Materials part)), below we will discuss only **1** in comparison with **3**. 

The vertical excitation energy, *ΔE^VE^*, of the lowest S_0_→S_1_ electronic transition calculated for **E**(S_0_) is 3.70 eV (335 nm, CC2/aug-cc-pVDZ). The photoexcitation of **1E**(S_0_) of one of those ππ* excited states populates eventually the lowest excited state (S_1_) at the Franck–Condon region what is indicated by a green dot in Figure 1a. Since *ΔE^VE^* for **1E** is lying above the potential energy profiles describing both processes, the *trans*–*cis* photoisomerization competes with the ESIPT process, leading toward the **1K_E_** tautomer (both indicated by the white arrow in Figure 1a). Both processes represent, in fact, a ballistic wave packet motion [29] along a given coordinate (either θ or R(OH), while α is practically not affected) without significant barriers and both can be easily activated upon photoexcitation of **E**(S_0_), since the adiabatic energies of their S_1_-state energy profiles are lying much below the vertical excitation energies, *ΔE^VE^*, of the absorbing ππ* states (compare Table 1, Table 2 and Table S1). 

As seen in Figure 1, a diagonal rim is representing the excited state barrier separating two deep S_1_-state minima. The proton-transferred **K_E_**(S_1_) form is at the upper-right corner (blue dot) and the **E_cis_**(S_1_) isomer is localized at the bottom-left corner (red dot). While the arrow, illustrating **E** to **E_cis_** isomerization, is almost parallel to the θ-axis, the arrow indicating the ESIPT process is tilted away from horizontal movement indicating the mixing of both the R(OH) and θ coordinates along the process. It is also evident that the *trans*–*cis* isomerization is a more exoergic process than the ESIPT one and should be a more preferred channel. It ends up at the CI(S_1_/S_0_) region for θ close to 90^o^. In the CI geometry, the internal conversion takes place, where the S_1_-state is depopulated to the ground state and the molecule can either populate the new **E_cis_**(S_0_) minimum or repopulate back the **E**(S_0_) isomer.

If the molecule undergoes the ESIPT process, leading towards the **K_E_**(S_1_) minimum (*E^a^* = 2.692 eV), the obtained **K_E_**(S_1_) form may emit E_fl_ = 1.4 eV or may further photoisomerize to the **K_K_** tautomer (see Figure 2). In a such photoisomerization reaction, the rotation of the protonated crane vs. the **7HQ** frame about the α dihedral angle is needed. A relatively low excited-state energy barrier should be overpassed (see the solid blue line in Figure 2a, left panel, for compound **1**, and in Figure 2b, for compound **3**) to reach the conical intersection region CI(S_1_/S_0_). This excited-state barrier is much lower than the S_0_-state barrier, where the rotation is around a double bond since the same double bond becomes nominally a single bond being elongated in the excited state. Along this excited-state process, related to the twist of the protonated crane vs. molecular **7HQ** frame, the molecule meets the conical intersection region CI(S_1_/S_0_) for the geometry, where the protonated crane and the frame are almost perpendicular to each other. The CI(S_1_/S_0_) geometry becomes a bifurcation point of the reaction, where depopulation to the ground state may favor either a return to the initial enol **E** form or a generation of a new **K_K_** tautomer. 

The shape of the excited-state energy profile along the N_1_H coordinate in **1** (Figure 2a, right panel) indicates the presence of both the ground and the excited-state energy barrier for the proton transfer from **K_K_**(S_1_) to **K**(S_1_), so these barriers also preclude direct population of the **K** form.

Comparing compounds **1** vs. **3,** one may notice that the S_1_-state minimum potential energy surfaces are also similar, indicating competition between two barrierless downhill processes: ESIPT and *trans*–*cis* photoisomerization. The excited-state proton-transferred minimum for **3** is a little more stable, *E^a^*=2.647 eV (see Figure 1b, details are given in the Supplementary Materials part Table S3), than that for **1** of *E^a^*=2.692 eV, and the OH distance in the excited state **K_E_** form is more elongate, being 2.040 A, for **3**, vs. 1.947 A, for **1**.

Bearing in mind that Figure 1 and Figure 2 originate from the gas phase calculational results, the transfer to the solution could change the relative stabilities of the excited-state species, making one or another pathway more favorable. In this respect, two points should be taken into account: the dipole moments of **K_E_** and **E_cis_** in S_1_, and the overstabilization of the keto tautomers by MP2-based calculations, as discussed above. A definite answer can be given by the experiment, but first, we should discuss what could be expected as a result of each of the competitive processes.

### 2.3. Long-Range PT and Trans–cis Isomerization in Terms of Spectral Changes to Be Experimentally Detected

The results in Table 1, Table 2 and Table S3 clearly suggest which kind of spectral changes could be expected upon ESIPT switching. The predicted absorption spectra of **1** are simulated in Figure 3a. It should be noted, that method-determined deviations from the experimental data exist in the absolute values, but in the discussion, the relative changes are of importance to show the expected tendencies [30]. According to the theoretically predicted absorption band positions, the appearance of the intermediate keto tautomers should cause a slight red shift in the absorption, while the appearance of the terminal **K** form leads to substantial bathochromic and hypochromic effects. As seen from the chemical structures of **1** and **2**, the intermediate keto tautomers (**K_E_** and **K_K_**) contain the same conjugated motif and their absorption spectra should be similar, which makes their detection practically impossible by using the optical spectra. More or less the same could be stated for **3**, but the steric hindrance in **K_K_** leads to lower oscillator strength, which could be used as a kind of proof if it appears. Actually, a very slight red shift in both absorption and emission was observed in the case of **1,** experimentally upon irradiation [24], leading to the conclusion that the **K_K_** form, but not **K**, is populated in agreement with the theoretical predictions. In the case of **3,** the irradiation does not cause any spectral changes [24] due to the instability of **K_K_** in the ground state, which leads to a reduced **K_K_***→***K_E_** barrier (~1.2 eV, see Table S2 in the Supplementary Materials part), and to the very fast thermal restoration of the equilibrium between **E** and **K_E_**, being separated by a very low barrier of +0.025 eV in respect of **K_E_**. 

As seen from the data in Table 1 and Figure 3a, the appearance of the *cis* enol (**E**_cis_) form could lead to hypochromic and hypochromic effects in the absorption spectra. In the case of a pure *trans*–*cis* transition, such an effect could be noticed as a reduction of the initial enol absorption, because the populated **E_cis_** should return relatively slowly to **E**. As a pure effect, it was not observed in **1** experimentally [24], but in general, blue shifts in the spectra are not easy to detect, especially if the content of the *cis* form is low and additional processes occur. More precisely this can be seen in **3**. The photoinitiated ESIPT, leading to a population only of **K_E_** in the ground state, cannot be detected by absorption spectroscopy, because the back relaxation to the existing thermal equilibrium is extremely fast. The *trans*–*cis* process should be detected, if occurring due to the significant barrier of the **E_cis_**→**E** process (see Table S2 in the Supplementary Materials part). The absorption spectra of **3** under irradiation do not change, which proves the appearance only of the tautomeric process.

Here is the moment to discuss the use of NMR for monitoring the long-range PT process. Due to the very low ground state energy barriers, the proton transfer between **E** and **K_E_,** and between **K_K_** and **K**, is too fast in the NMR time scale [31]. For this reason, averaged signals of the pair **E**/**K_E_** and **K_K_**/**K** could be only detected. At the same time, the participation of the tautomeric proton in strong intramolecular hydrogen bonding in any of four possible tautomeric forms leads to a down-fielded ^1^H chemical shift, much above 10 ppm. The observed chemical shifts depend on the population of the particular tautomer in the pair and its individual chemical shift [32]. As it is sketched in Figure 3b the ranges of signals of the pairs **E**/**K_E_** and **K_K_**/**K** are given in pink and light blue, correspondingly. Comparing only the terminal **E** and **K** tautomers, in general, the NH chemical shift of the **K** form should be substantially down-fielded in comparison with the OH shift of the enol (Figure 3b). In the case of **1**, the existence of two NH signals is detected in CD_3_CN, a strong intensive at 15.47 and a weak one at 14.25 ppm. The former is a result of the co-existence of the tautomers in the pair **E**/**K_E_**, while the latter is attributed to **K_K_** and can be used for monitoring the switching process upon irradiation. The **1K** tautomer, which NH chemical shift should be measured > 17 ppm, is impossible to appear due to the low stability as discussed above, which is the reason for the up-field (from 15.47 to 14.25) of the signal following the internal rotation around the axle.

The tautomeric behavior of **2** is substantially easier to interpret. The fluorine substitution, compared to **1**, leads to reduced basicity of the azomethine nitrogen atom and, hence, to the lower stability of the intermediate tautomer. The predominance of **E** and the existence of traces only on **K_E_** only is evident from the absorption and NMR spectra in full agreement with the data, collected in Table S1 (See Supplementary Materials Part). According to the photochemical experiments, the irradiation leads to a slight red shift in both absorption and emission spectra, indicating populating of the **K_K_** tautomer as a result. As it happens in **1**, no direct evidence for the *trans*–*cis* process is found, but the difficulties in detecting the blue-shifted **E_cis_** should be taken into account in this respect. 

In all studied compounds, the yield of the aimed ESIPT process is reduced, even if **E_cis_** is not directly populated, as a result of the *trans*–*cis* isomerization in the excited state, i.e., the relaxation through CI leads only back to **E**. This might be an explanation for the observed low efficiency of ESIPT in the Schiff bases [24,33]. 

## 3. Theoretical Methodology

Theoretical calculations were performed in vacuo without imposing any symmetry constraints. The ground state (S_0_) of the molecules is modeled with the MP2 method [34] with the use of the correlation-consistent valence double zeta basis set with polarization functions on all atoms (cc-pVDZ) [35]. The MP2/cc-pVDZ energy of the enol form **E** is the reference energy for other isomers of the molecules, both in the ground and in the excited state. The same method was used to estimate the potential energy barriers separating the isomers in their ground state. The effect of using aug-cc-pVDZ and cc-pVTZ basis sets, shown in Table S4 (see Supplementary Materials part), indicates that the change of the basis set does not lead to a dramatic change in the overall PES and relative stabilities of the tautomers, proving the use of the less computationally demanding cc-pVDZ.

The UV spectrum was theoretically simulated with aid of the CC2 [36,37] method with the aug-cc-pVDZ basis set [35] and with the ADC(2) method [38,39,40,41] for comparison. ADC(2) is a computationally efficient single-reference propagator method, which yields similar excitation energies as the simplified second-order coupled-cluster (CC2) method [36]. Additionally, the ADC(2) energy for the ground state is equal to the MP2 energy (MP2 energy is a reference for the ADC(2) method). The accuracy of CC2 and ADC(2) for excitation energies of organic molecules has been extensively benchmarked in comparison with more accurate methods, such as CC3 and EOM-CCSD [42,43,44].

Certain problems were experienced with the choice of the proper method for excited-state optimization. The CC2/cc-pVDZ method gives barrierless ESIPT from the photoexcited S_0_-state **E** global minimum in its Franck–Condon region toward the proton-transferred **K_E_** tautomer, while the optimization with the ADC(2)/cc-pVDZ method shows barrierless evolution of the same **E** form toward the **E_cis_** isomer of the molecule. However, optimization of the ADC(2) and CC2 methods with larger basis sets aug-cc-pVDZ and cc-pVTZ finally converged to the **E_cis_** isomer. Such results give the idea that the processes photoinitiated at the **E** form (ESIPT and *trans*-*cis* photoisomerization), are competitive with each other and the best choice would be to present this competition in a form of the relaxed PES spanned over two coordinates: R(O-H) stretching and θ (C=N) torsion angle. For this purpose, the excited-state (S_1_) equilibrium geometries were determined with the second-order algebraic diagrammatic construction ADC(2) method [38,39,41], since this method seemed to be faster for relaxed energy surface calculations. The same method was used to estimate the S_1_-state energy barriers along the proton transfer: OH and NH stretching coordinates as well as for the torsion angles α and θ in a single parameter-relaxed potential energy scan calculation. All calculations were performed using the TURBOMOLE program package [45].

The absorption spectra were visualized in Figure 3a by using the Gauss shape [46] of the individual bands with a half-band width of 3000 cm^−1^ and molar absorptivity extracted from the predicted oscillator strength [47]. The NMR chemical shieldings of the tautomeric forms of the studied compounds were calculated using the GIAO approximation [48] using MP2/ cc-pVDZ. The calculated absolute shieldings were transformed to chemical shifts using the reference compound tetramethylsilane, Si(CH_3_)_4_, for the hydrogen atoms.

## 4. Conclusions

In the proton cranes, being Schiff bases derived from 7-hydroxyquinoline, two competitive channels are possible upon photoexcitation, namely proton transfer and *trans*–*cis* isomerization around the azomethine double bond in the enol tautomer. The former leads to switching, based on twist-assisted ESIPT, where the long-range proton transfer can occur as a targeted process. The latter, determined by the flexibility of the crane part, reduces the efficiency of the switching process. Both ESIPT and *trans*–*cis* photoisomerization are barrierless processes initiated from the **E**(S_0_) Franck–Condon region. Which of these two channels dominate depends on the chemical structure and the chemical environment, but the experimental spectral data allow us to recognize it. The tautomeric proton transfer leads to a red shift in the absorption spectra, while the *trans*–*cis* isomerization causes a hypochromic and hypochromic effect, originating from the reduced conjugation.

## Figures and Tables

**Table 1:** Vertical excitation energy, *ΔE^VE^* (in eV), oscillator strength, *f*, and dipole moment, *μ_e_ (*in Debye), of the lowest excited singlet states calculated with the CC2/aug-cc-pVDZ method for the ground state equilibrium forms of **1**, optimized at the MP2/cc-pVDZ theory level. The dipole moment of the ground-state, *μ_g_* (in Debye, MP2/cc-pVDZ).

Tautomer		*ΔE^VE^*	*f*	*μ_e_*

**1E** 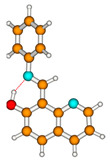	S_0_	*0.00*		*μ_g_* = 3.2
	S_0_→S_1_(ππ*)	3.70	0.344	0.7
	S_0_→S_2_(ππ*)	4.11	0.129	2.7
	S_0_→S_3_(ππ*)	4.32	0.235	2.1
	S_0_→S_4_(nπ*)	4.40	0.004	1.2
	S_0_→S_5_(ππ*)	4.58	0.020	1.8
	S_0_→S_6_(nπ*)	4.75	0.000	1.9

**1K_E_** 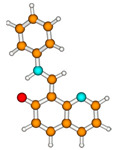	S_0_	*0.203* ^a^		*μ_g_* = 2.5
	S_0_→S_1_(ππ*)	3.14	0.515	2.4
	S_0_→S_2_(nπ*)	3.40	0.000	1.7
	S_0_→S_3_(ππ*)	3.89	0.090	5.3
	S_0_→S_4_(ππ*)	4.28	0.298	4.7
	S_0_→S_5_(nπ*)	4.47	0.000	7.6
	S_0_→S_6_(ππ*)	4.61	0.022	2.9

**1K_K_** 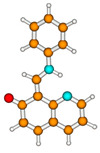	S_0_	*0.274* ^a^		*μ_g_* = 4.7
	S_0_→S_1_(ππ*)	3.04	0.521	5.6
	S_0_→S_2_(nπ*)	3.15	0.001	0.9
	S_0_→S_3_(ππ*)	3.72	0.128	5.1
	S_0_→S_4_(ππ*)	4.35	0.207	6.1
	S_0_→S_5_(ππ*)	4.56	0.029	5.3
	S_0_→S_6_(nπ*)	4.57	0.000	8.2

**1K** 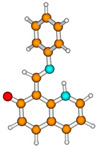	S_0_	*0.535* ^a^		*μ_g_* = 7.3
	S_0_→S_1_(ππ*)	2.81	0.109	5.4
	S_0_→S_2_(nπ*)	3.09	0.001	1.9
	S_0_→S_3_(ππ*)	3.38	0.488	6.1
	S_0_→S_4_(nπ*)	3.84	0.001	3.8
	S_0_→S_5_(ππ*)	4.33	0.028	7.0
	S_0_→S_6_(nπ*)	4.43	0.004	5.6

**1E_cis_** 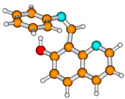	S_0_	*0.531* ^a^		*μ_g_* = 3.9
	S_0_→S_1_(ππ*)	3.93	0.086	2.8
	S_0_→S_2_(ππ*)	4.13	0.081	3.4
	S_0_→S_3_(ππ*)	4.42	0.038	2.2
	S_0_→S_4_(nπ*)	4.55	0.024	3.2
	S_0_→S_5_(ππ*)	4.66	0.052	4.1
	S_0_→S_6_(nπ*)	4.80	0.048	4.0

^a—^*E^a^*, relative to the **1E** form optimized at the MP2/cc-pVDZ theory level.

**Table 2:** Vertical excitation energy, Δ*E^VE^* (in eV), oscillator strength, *f*, and dipole moment, *μ_e_ (*in Debye), of the lowest excited singlet states calculated with the CC2/aug-cc-pVDZ method for the ground state equilibrium forms of **3**, optimized at the MP2/cc-pVDZ theory level. The dipole moment of the ground-state, *μ_g_* (in Debye, MP2/cc-pVDZ).

Tautomers		*ΔE^VE^*	*f*	*μ_e_*

**3E** 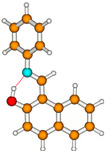	S_0_	*0.00*		*μ_g_* = 2.4
	S_0_→S_1_(ππ*)	3.56	0.373	3.0
	S_0_→S_2_(ππ*)	4.13	0.214	2.8
	S_0_→S_3_(nπ*)	4.36	0.003	3.0
	S_0_→S_4_(nπ*)	4.46	0.001	0.9
	S_0_→S_5_(nπ*)	4.74	0.001	2.6
	S_0_→S_6_(ππ*)	4.95	0.098	0.3

**3K_E_** 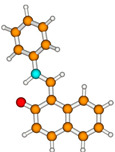	S_0_	*0.240* ^a^		*μ_g_* = 3.0
	S_0_→S_1_(ππ*)	3.05	0.487	3.5
	S_0_→S_2_(nπ*)	3.37	0.001	1.6
	S_0_→S_3_(ππ*)	3.94	0.027	4.2
	S_0_→S_4_(ππ*)	4.15	0.253	3.3
	S_0_→S_5_(nπ*)	4.56	0.018	3.5
	S_0_→S_6_(nπ*)	4.66	0.001	7.1

**3K_K_** 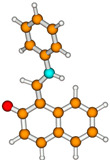	S_0_	*0.611* ^a^		*μ_g_* = 4.6
	S_0_→S_1_(ππ*)	3.07	0.269	4.6
	S_0_→S_2_(nπ*)	3.21	0.185	3.4
	S_0_→S_3_(ππ*)	3.98	0.005	3.7
	S_0_→S_4_(ππ*)	4.29	0.319	4.9
	S_0_→S_5_(ππ*)	4.53	0.017	4.6
	S_0_→S_6_(nπ*)	4.58	0.025	4.4

**3E_cis_** 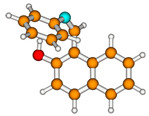	S_0_	*0.450* ^a^		*μ_g_* = 2.0
	S_0_→S_1_(ππ*)	3.82	0.107	2.8
	S_0_→S_2_(ππ*)	4.16	0.092	3.4
	S_0_→S_3_(ππ*)	4.39	0.033	4.2
	S_0_→S_4_(nπ*)	4.62	0.021	2.6
	S_0_→S_5_(ππ*)	4.74	0.001	1.5
	S_0_→S_6_(nπ*)	5.14	0.175	5.6

^a—^*E^a^*, relative to the **3E** form optimized at the MP2/cc-pVDZ theory level.

## Data Availability

The data presented in this study are available in this article.

## Supplementary Materials

The following supporting information can be downloaded at: https://www.mdpi.com/article/10.3390/molecules27238225/s1, Figure S1: Potential energy profiles of **2** in the S_0_ state (circles), determined at the MP2/cc-pVDZ theory level, in the S_1_ state (blue squares), determined at the ADC(2)/cc-pVDZ theory level along the minimum-energy path for N-phenyl crane torsion (**K_E_** to **K_K_**), and for hydrogen transfer from the intermediate form **K_K_**, toward proton-transferred form, **K**. S_0_(S_1_) denotes the energy of the S_0_ state, calculated along the minimum energy path of the S_1_ excited state (red dashed curves); Table S1: Vertical excitation energy, *ΔE^VE^* (in eV), oscillator strength, *f*, and dipole moment, *μ_e_ (*in Debye), of the lowest excited singlet states calculated with the CC2/aug-cc-pVDZ method for the ground state equilibrium forms of **2**, optimized at the MP2/cc-pVDZ theory level; Table S2: Relative S_0_-state adiabatic energies, *E^a^*, (in eV), of the stable tautomers and S_0_-state energy barriers separating the ground-state minima of the studied compounds **1**, **2** and **3**; Table S3: Adiabatic energy, *E^a^*, and fluorescence energy, E_fl_, in eV, excited-state dipole moment, *μ_e_*, in Debye, and OH distance for different excited-state minima of compounds **1**, **2** and **3**; Table S4: Ground-state S_0_ energy minima (relative energy in eV) and energy barriers of compound **1** optimized with the MP2 method using three different basis sets: cc-pVDZ, cc-pVTZ and aug-cc-pVDZ.
