# Supplementary material for: Long-Range Proton Transfer in 7-Hydroxy-Quinoline-Based Azomethine Dyes: A Hidden Reason for the Low Efficiency
Source: Molecules. 2022 Nov 25;27(23):8225. doi: 10.3390/molecules27238225 (PMC9736288; doi:10.3390/molecules27238225)
Supplement: Supplementary file 1 [file molecules-27-08225-s001.zip › molecules-2007194-supplementary.pdf]

Supplementary Information

Long-range proton transfer in 7-hydroxy-quinoline based azomethine dyes: a hidden reason for the low efficiency

Michał F. Rode [1,*], Daniela Nedeltcheva[2,3] and Liudmil Antonov[2,*]

[1] Institute of Physics, Polish Academy of Sciences, Aleja Lotników 32/46, 02-668 Warsaw, Poland

[2] Institute of Electronics, Bulgarian Academy of Sciences, 72 Tzarigradsko chaussee blvd., Sofia 1784, Bulgaria

3 Institute of Organic Chemistry with Centre of Phytochemistry, Acad. G.Bonchev str., bl. 9, Sofia 1113, Bulgaria

Table S1. Vertical excitation energy, ΔE^{VE} (in eV), oscillator strength, f, and dipole moment, μ_e (in Debye), of the lowest excited singlet states calculated with the CC2/aug-cc-pVDZ method for the ground state equilibrium forms of **2**, optimized at the MP2/cc-pVDZ theory level. Dipole moment of the ground-state, μ_g (in Debye, MP2/cc-pVDZ).

Tautomers		ΔE^{VE}	f	μ_e
**2E** 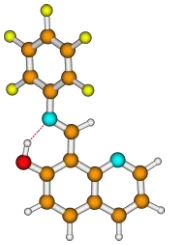	S_0	*0.00*		μ_g = 6.0
	$S_0 \rightarrow S_1(\pi\pi^*)$	3.67	0.359	5.4
	$S_0 \rightarrow S_2(\pi\pi^*)$	4.12	0.141	3.7
	$S_0 \rightarrow S_3(\pi\pi^*)$	4.30	0.260	6.2
	$S_0 \rightarrow S_4(n\pi^*)$	4.43	0.003	4.1
	$S_0 \rightarrow S_5(n\pi^*)$	4.60	0.003	2.6
	$S_0 \rightarrow S_6(n\pi^*)$	4.72	0.006	1.5
**$2K_E$** 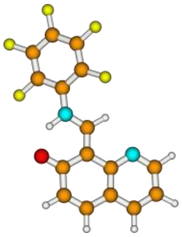	S_0	*0.243* [a]		μ_g = 4.4
	$S_0 \rightarrow S_1(\pi\pi^*)$	3.20	0.506	3.5
	$S_0 \rightarrow S_2(n\pi^*)$	3.37	0.000	3.6
	$S_0 \rightarrow S_3(\pi\pi^*)$	4.00	0.029	1.0
	$S_0 \rightarrow S_4(\pi\pi^*)$	4.26	0.469	5.9
**$2K_K$** 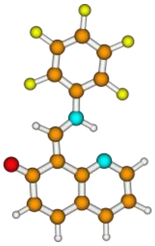	S_0	*0.282*[a]		μ_g = 5.6
	$S_0 \rightarrow S_1(n\pi^*)$	3.10	0.000	
	$S_0 \rightarrow S_2(\pi\pi^*)$	3.16	0.547	
	$S_0 \rightarrow S_3(\pi\pi^*)$	3.84	0.091	
	$S_0 \rightarrow S_4(\pi\pi^*)$	4.37	0.287	
	$S_0 \rightarrow S_5(n\pi^*)$	4.49	0.001	
	$S_0 \rightarrow S_6(n\pi^*)$			
**2K** 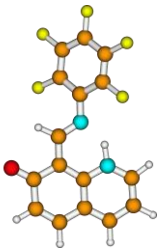	S_0	*0.472*[a]		μ_g = 8.8
	$S_0 \rightarrow S_1(\pi\pi^*)$	2.93	0.130	3.7
	$S_0 \rightarrow S_2(n\pi^*)$	3.11	0.001	2.6
	$S_0 \rightarrow S_3(\pi\pi^*)$	3.23	0.508	8.1
	$S_0 \rightarrow S_4(\pi\pi^*)$	3.73	0.000	6.9
	$S_0 \rightarrow S_5(\pi\pi^*)$	4.44	0.042	4.2
	$S_0 \rightarrow S_6(n\pi^*)$	4.52	0.002	9.3
**$2E_{cis}$**	S_0	*0.436*[a]		μ_g = 5.5
	$S_0 \rightarrow S_1(\pi\pi^*)$	3.99	0.075	4.0

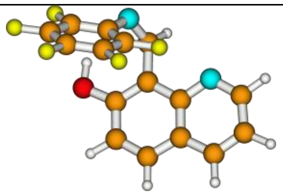	$S_0 \rightarrow S_2(\pi\pi^*)$	4.25	0.062	1.2
	$S_0 \rightarrow S_3(\pi\pi^*)$	4.45	0.031	4.0
	$S_0 \rightarrow S_4(n\pi^*)$	4.59	0.094	5.8
	$S_0 \rightarrow S_5(\pi\pi^*)$	4.70	0.009	4.8
	$S_0 \rightarrow S_6(n\pi^*)$	4.88	0.018	3.8

[a] – E^a, relative to the **3E** form optimized at the MP2/cc-pVDZ theory level.

Tables S2. Relative S_0-state adiabatic energies, E^a, (in eV), of the stable tautomers and S_0-state energy barriers (***in italic***) separating the ground-state minima of the studied compounds **1**, **2** and **3**.

**Compound 1**								
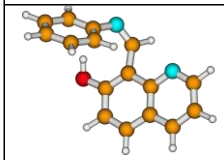		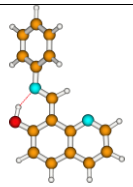		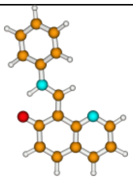		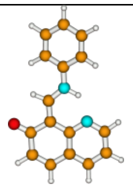		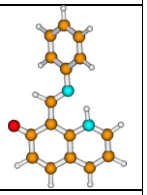
Form: **E_{cis}**		**E**		**K_E**		**K_K**		**K**
E^a = 0.531	1.351	0.00	0.251	0.203	1.850	0.273	0.595	0.535
Barrier	***+0.82***		***+0.048***		***+1.6***		***+0.60***	
**Compound 2**								
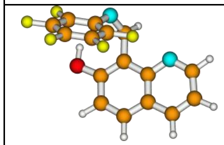		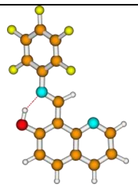		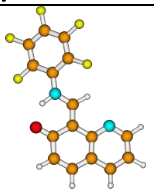		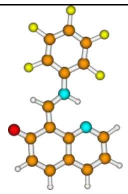		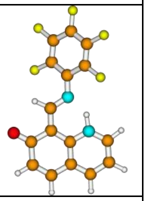
Form: **E_{cis}**		**E**		**K_E**		**K_K**		**K**
E^a = 0.436	1.360	0.00	0.280	0.243	1.970	0.282	0.530	0.472
Barrier	***+0.93***		***+0.037***		***+1.7***		***+0.58***	
**Compound 3**								
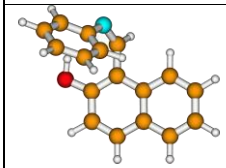		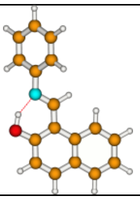		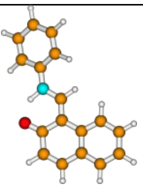		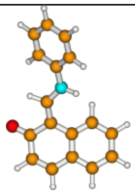		
Form: **E_{Cis}**		**E_{Trans}**		**K_E**		**K_K**		
E^a **0.450**	1.310	0.00	0.265	0.240	1.810	0.611		
Barrier	***+0.86***		***+0.025***		***+1.2***			

Table S3. Adiabatic energy, E^a, and fluorescence energy, E_{fl}, in eV, excited-state dipole moment, μ_e, in Debye, and OH distance for different excited-state minima of compounds **1**, **2** and **3**.

proton-transferred excited-state minimum $\mathbf{K_E}(S_1)$			
	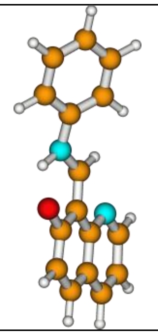	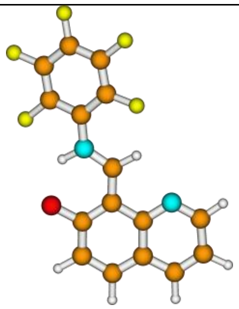	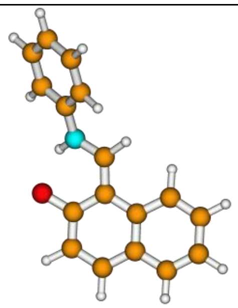
**Compound**	**1**	**2**	**3**
E^a, eV	2.692	2.677	2.647
E_{fl}, eV	1.40	1.40	1.08
μ_e, D	3.2	5.0	2.9 D
OH, Å	1.947	1.901	2.040

Excited-state minimum $\mathbf{E_{Cis}}(S_1)$			
	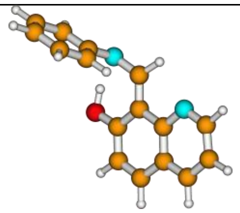	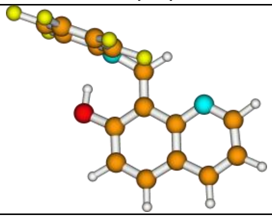	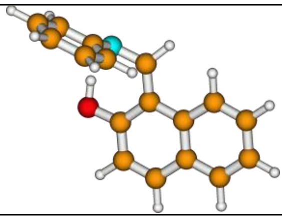
**Compound**	**1**	**2**	**3**
E^a, eV	2.329	2.544	2.287
E_{fl}, eV	-0.064	0.38	-0.033
μ_e, D	2.7	1.55	3.3
θ	86.0°	86.1°	87.2°

Table S4. Ground-state S_0 energy minima (relative energy in eV) and energy barriers of compound **1** optimized with the MP2 method using three different basis sets: cc-pVDZ, cc-pVTZ and aug-cc-pVDZ. Dipole moments are in Italic.

Method	E(S_0)	TS_1	$K_E(S_0)$	TS_2	$K_K(S_0)$	TS_3	K(S_0)	$E_{cis}(S_0)$
**S_0 state for 1**								
**MP2/cc-pVDZ**	0.00 *3.2 D*	0.251	0.203 *2.5 D*	1.850	0.273 *4.0 D*	0.595	0.535 *6.0 D*	0.531 *3.9 D*
**S_0-state barrier**		+0.48		+1.647		+0.060		
**MP2/cc-pVTZ**	0.00	0.223	0.173 *2.5 D*		0.278 *4.8 D*		0.525 *7.4 D*	0.485 *3.9 D*
**S_0-state barrier**		+0.49						
**MP2/ aug-cc-pVDZ**	0.00 *3.3 D*	0.209	0.149 *2.7 D*	1.700	0.253 *5.0 D*	0.555	0.487 *7.6 D*	0.367 *4.0 D*
**S_0-state barrier**		+0.60		+1.509		+0.068		

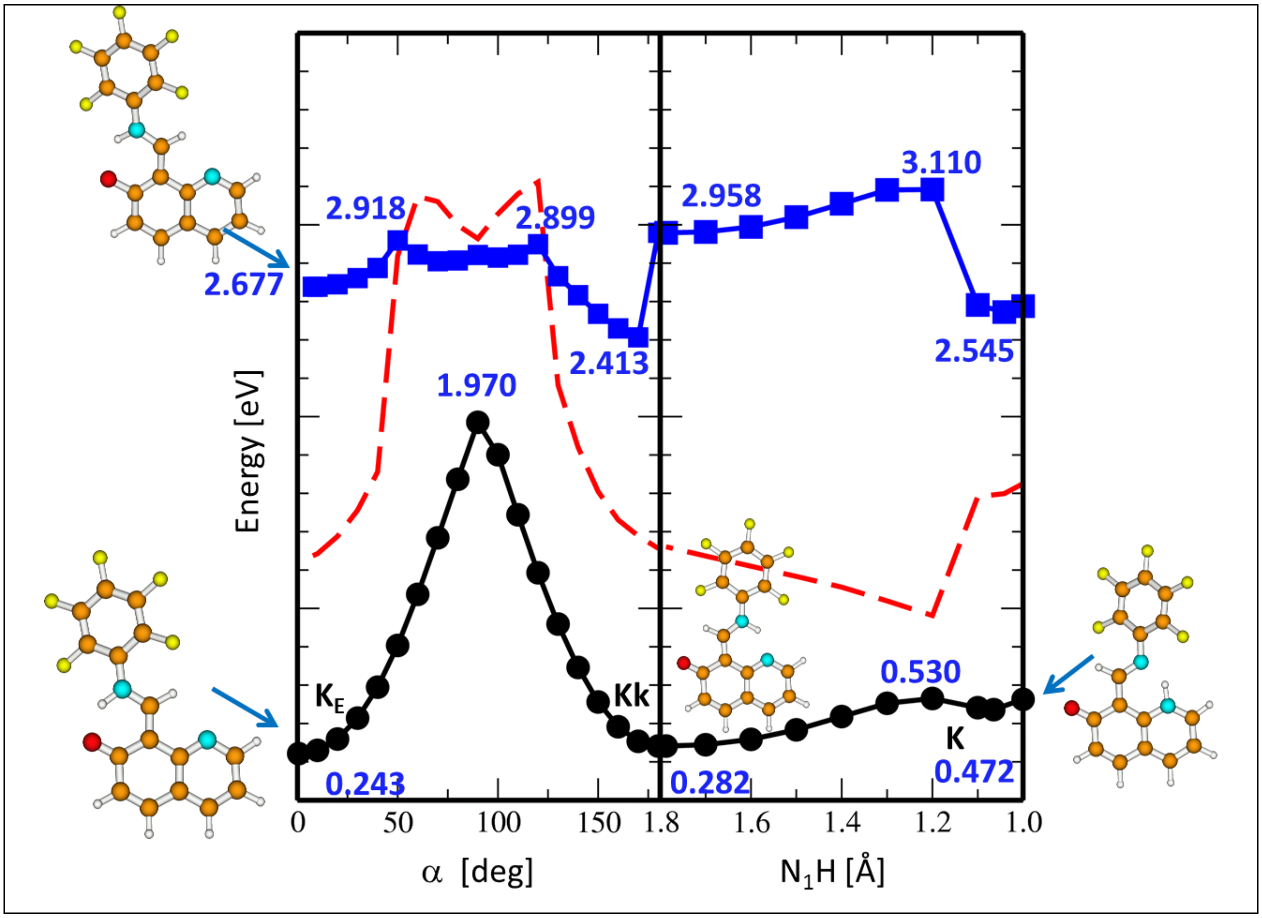

Figure S1. Potential energy profiles of **2** in the S_0 state (circles), determined at the MP2/cc-pVDZ theory level, in the S_1 state (blue squares), determined at the ADC(2)/cc-pVDZ theory level along the minimum-energy path for N-phenyl crane torsion (**K_E** to **K_K**), and for hydrogen transfer from the intermediate form **K_K**, toward proton-transferred form, **K**. $S_0(S_1)$ denotes the energy of the S_0 state, calculated along the minimum energy path of the S_1 excited state (red dashed curves).
